# Evidence for influx of Atlantic water masses to the Labrador Sea during the Last Glacial Maximum

**DOI:** 10.1038/s41598-021-86224-z

**Published:** 2021-03-24

**Authors:** Marit-Solveig Seidenkrantz, Antoon Kuijpers, Steffen Aagaard-Sørensen, Holger Lindgreen, Jesper Olsen, Christof Pearce

**Affiliations:** 1 Paleoceanography and Paleoclimate Group, Arctic Research Centre, and iCLIMATE Aarhus University Interdisciplinary Centre for Climate Change, Department for Geoscience, Aarhus University, Høegh-Guldbergs Gade 2, 8000 Aarhus C, Denmark; 2 Geological Survey of Denmark and Greenland (GEUS), Øster Voldgade 10, 1350 Copenhagen K, Denmark; 3 Department of Geosciences, UiT The Arctic University of Norway in Tromsø, Postboks 6050 Langnes, 9037 Tromsø, Norway; 4 Aarhus AMS Centre, Department of Physics and Astronomy, Aarhus University, Ny Munkegade 120, 8000 Aarhus C, Denmark

**Keywords:** Palaeoceanography, Palaeoclimate, Climate sciences, Ocean sciences

## Abstract

The Last Glacial Maximum (LGM, 23–19,000 year BP) designates a period of extensive glacial extent and very cold conditions on the Northern Hemisphere. The strength of ocean circulation during this period has been highly debated. Based on investigations of two marine sediment cores from the Davis Strait (1033 m water depth) and the northern Labrador Sea (2381 m), we demonstrate a significant influx of Atlantic-sourced water at both subsurface and intermediate depths during the LGM. Although surface-water conditions were cold and sea-ice loaded, the lower strata of the (proto) West Greenland Current carried a significant Atlantic (Irminger Sea-derived) Water signal, while at the deeper site the sea floor was swept by a water mass comparable with present Northeast Atlantic Deep Water. The persistent influx of these Atlantic-sourced waters entrained by boundary currents off SW Greenland demonstrates an active Atlantic Meridional Overturning Circulation during the LGM. Immediately after the LGM, deglaciation was characterized by a prominent deep-water ventilation event and potentially Labrador Sea Water formation, presumably related to brine formation and/or hyperpycnal meltwater flows. This was followed by a major re-arrangement of deep-water masses most likely linked to increased overflow at the Greenland-Scotland Ridge after ca 15 kyr BP.

## Introduction

The large ice sheets that surrounded the North Atlantic during the Last Glacial Maximum (LGM; 23,000–19,000 years ago (23–19 kyr BP, before present))^1^ together with a presumably widespread sea-ice cover^2^ had a significant influence on regional oceanic conditions. LGM ocean circulation has been subject to extensive studies based on both proxy data and models^3, 4^. There are notably large discrepancies in results from different model simulations ranging from significantly colder to anomalously warm LGM sea-surface conditions over parts of the North Atlantic^5^. As deep convection processes are controlled by ocean cooling and freezing processes as well as wind stress and temperature-dependent evaporation, the impact of the presence of the large LGM ice sheets on prevailing ocean and atmosphere circulation is crucial for understanding apparently persisting deep-water formation in the North Atlantic during the LGM^4^.

Today North Atlantic deep-water formation occurs in the Nordic Seas and the North Atlantic Subpolar Gyre (SPG) region^6, 7^. For the SPG region, the most important deep mixing centre is found in the Labrador Sea^8, 9^, although deep convection has also been reported from the Irminger Sea^10, 11^. However, the location and strength of North Atlantic deep convection during the last glacial period has been debated. Previously, it was suggested that the main North Atlantic deep convection occurred south of Iceland during the LGM, with the deeper waters only reaching intermediate levels producing Glacial North Atlantic Intermediate Water (GNAIW)^12, 13^. More recently, evidence was put forward for deep water masses formed in the SPG during the LGM^14^. Other studies indicate that a sustained production of North Atlantic Deep Water (NADW) took place in the North Atlantic also under glacial conditions^4^ and that deep-water formation occurred in the Norwegian Sea, too^15^. However, deep convection in the Nordic Seas during the LGM was likely weak and unstable^16^, resulting in an only intermittent overflow across the Greenland–Scotland Ridge^17^. Not until the onset of the Bølling–Allerød climate warming at ~ 15 kyr BP, a stronger and more stable deep convection was likely initiated in the Nordic Seas, as is evident from persistent overflow across the Greenland-Scotland Ridge^18^.

Deep and surface circulation in the SPG and Labrador Sea are very important elements of the Atlantic Meridional Overturning Circulation (AMOC) cell and consequently global ocean circulation. Formation of Labrador Sea Water (LSW) contributing to (upper) NADW has, however, been found to show large variability throughout the past decades^9, 19–21^. During the LGM, the Labrador Sea is believed to have been characterised by cold sea-surface temperatures and perennial sea-ice cover along the Canadian and Greenland margins, while the central North Atlantic and Norwegian Sea may have been seasonally ice-free^2^. However, the implications of this scenario for deep-water circulation in the North Atlantic and potential deep convection in the Labrador Sea during the LGM are yet uncertain.

**Figure 1:**
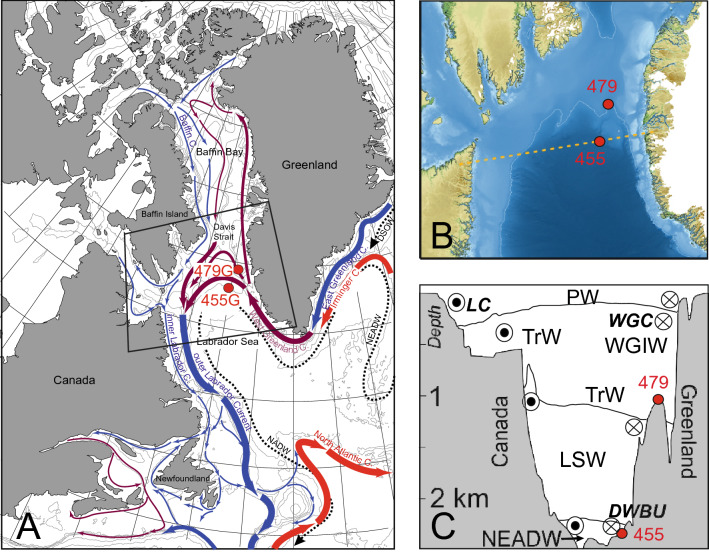
Regional setting for sediment gravity cores TTR13-AT-479G (labelled 479G) located at 64°24.37′ N, 55°45.08′ W (1033 m present water depth), and TTR13-AT-455G (labelled 455G) located at 62°52,17′ N; 55°11,22′ W (2381 m water depth). (**A**) The modern surface ocean circulation pattern (modified after^32, 89^) (red: warm surface currents; blue: cold surface currents; purple: cool current formed as a mixture of warmer and cold currents; black hatched: NADW = North Atlantic Deep Water, NEADW = Northeast Atlantic Deep Water and DSOW = Denmark Strait Overflow Water). LC = Labrador Current. WGC = West Greenland current. (**B**) Detailed map of the study region combined from GEBCO^90^ and BedMachine^91^ data. It illustrates that core 479G is located on a plateau, while core 455G is located in the basin not far away from the mouth of a major canyon (Fylla Canyon^25^). The orange hatched line designates the track of the hydrographic transect shown in (**C**). (**C**) Simplified vertical distribution of the water masses in the study region modified from World Ocean Atlas^92^. LC = Labrador Current; WGC = West Greenland Current; DWBU = Deep Western Boundary Undercurrent; PW = Polar Water; WGIW = West Greenland Irminger Water (Atlantic-soured water); TrW = Transitional Water, where the Atlantic-source WGIW is partly mixed with PW; LSW = Labrador Sea Water; NEADW = Northeast Atlantic Deep Water. Note that LSW and NEADW is only found in the northern Labrador Sea, not in the Davis Strait. For further details see supplementary Fig. S1.

We here aim to improve our understanding of thermohaline responses in the Labrador Sea to the fundamentally different LGM boundary conditions and the implications for the AMOC. Our study is based on surface, subsurface and intermediate/deep water information derived from planktic and benthic foraminiferal assemblages, supported by sedimentological and geochemical proxies from two marine sediment cores collected at 2381 and 1033 m water depth in the northernmost Labrador Sea and Davis Strait, respectively (Fig. 1). Benthic foraminifera are highly sensitive to changes in bottom-water conditions and thus are excellent indicators of past environments and changes in ocean circulation, while planktic foraminifera reflect surface-water conditions. As our study is based on the comparison to present-day oceanographic conditions, we here use the modern water mass terminologies.

### Modern hydrography of the Labrador Sea and the Davis Strait

The modern large-scale ocean surface circulation pattern of the Labrador Sea is characterised by fast boundary currents. The West Greenland Current (WGC; Fig. 1) transports Atlantic-sourced water masses from the Irminger Sea (West Greenland Irminger Water, WGIW; Figs. 1, S1) into the northern Labrador Sea, Davis Strait, and eastern Baffin Bay at subsurface depths^22^. The upper part of the WGC consists of a comparatively thin (< ca. 150 m) layer of cold, low-salinity Polar Water derived from the East Greenland Current and melt water discharged from local Greenland outlet glaciers (Fig. 1), overlying the relatively warm Atlantic-source subsurface water masses^23^. The influx of both the Atlantic and Polar water masses to the Labrador Sea is linked to the strength and position of the North Atlantic SPG^24^, with the present day advection of warm and saline WGIW being at maximum strength with highest temperatures, salinities and velocities during winter, when also the transport of Atlantic-sourced water via the Irminger Current is at a maximum^25^. Current strength in the study region may however vary significantly due to local or regional topographic effects^26^. Part of the warmer and more saline WGIW water masses reaches the interior of the Labrador Sea basin south of the Davis Strait as a weak, eddy-dominated flow^22^. This flow of saline water to the central Labrador Sea is crucial for the formation of Labrador Sea Water (LSW), a deep to intermediate water mass contributing to upper NADW^9, 19^. However, a part of the relatively warm and saline Atlantic-sourced subsurface waters (WGIW) continues with the WGC northward along the shelf and upper slope of West Greenland across Davis Strait and into the Baffin Bay^27, 28^. In the Davis Strait, the WGIW dominates the slope off West Greenland, overlying a transitional water mass (TrW), which also separates the core of the WGIW from the cold and less saline Polar Water (PW) to the west^27^.

The deep-water circulation in the Labrador Sea basin is dominated by the Deep Western Boundary Undercurrent (DWBU), an important component of the North Atlantic thermohaline circulation cell^6^. The DWBU water masses in the deep Labrador basin are primarily comprised of dense Denmark Strait Overflow Water (DSOW; also called Northwest Atlantic Bottom Water), as well as Northeast Atlantic Deep Water (NEADW; Fig. 1). The dense DSOW masses underlie NEADW, which originates from mixing processes in the Iceland basin involving Iceland-Scotland Overflow Waters (ISOW). Both DSOW and NEADW together form the North Atlantic Deep Water (NADW). The DWBU flow, transporting NADW into the Labrador Sea basin, displays a strong, barotropic current core flowing above the lower, continental slope close to the 3000 m isobath^22, 29^. Along the southwest Greenland lower continental slope and rise, DWBU transport increases northward towards Davis Strait, where the current turns west. Increased current activity^30^ is associated with the presence of re-circulating deep waters and an increased volume of the denser bottom water classes. Another, shallower current maximum not directly related to the DWBU, is found higher up the southwest Greenland continental slope at the depth stratum between 1000 and 2000 m isobaths^22^. Neither the LSW nor the NEADW penetrates northwards into the Davis Strait^27^ due to the relatively shallow depth of the strait (Fig. S1).

### Study area and material

The sediment cores TTR13-AT-455G (hereafter 455G) and TTR13-AT-479G (hereafter 479G) were collected in 2003 during a cruise of RV *Prof. Logachev*^31^ from the northernmost Labrador Sea (455G: 62°52,17′ N; 55°11,22′ W; 2381 m water depth) and the south-eastern Davis Strait (479G: 64°24.37′ N 55°45.08′ W, 1033 m water depth; note slight correction of position compared to previously shown^32^), respectively (Fig. 1). Sediment core 479G has previously been presented^32^, whereas 455G data are presented here for the first time. Surface water conditions at both sites are today governed by Polar Waters with Atlantic-sourced (WGIW) water from the WGC found at subsurface depths. Bottom-water conditions at core 479G thus corresponds to the lowermost part of the WGC, i.e. the boundary zone between WGIW and TrW. In contrast, the sea floor at core 455G is flushed by NEADW and at times of extreme deep convection possibly also by LSW. Core 455G is located not far from the outlet of a major underwater canyon originating from the Greenland shelf edge further to the north, which may lead to conditions of possibly occasional flushing of the site by dense winter water formed on the shelf^26, 33^.

## Results

### Age model

**Figure 2:**
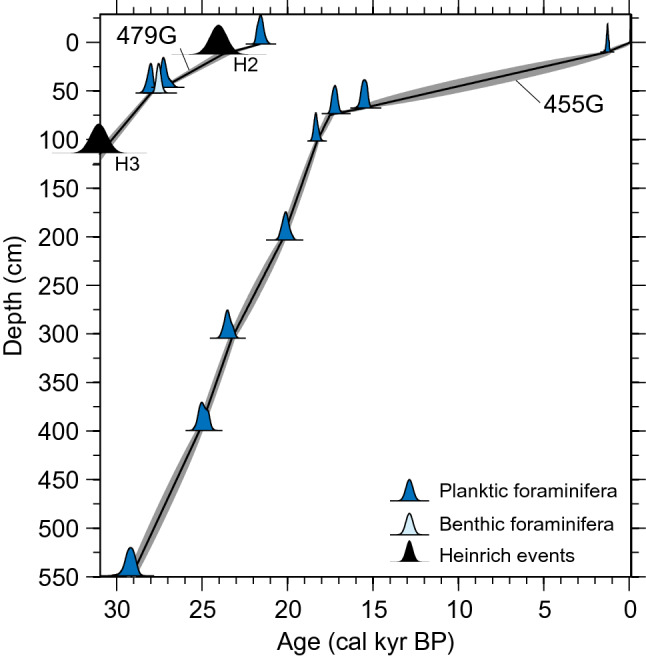
Age models of core TTR13-AT-455G and the top of core TTR13-AT-479G. The age model of core 455G is based on eight calibrated AMS ^14^C datings on the planktic foraminifera *Neogloboquadrina pachyderma* (sinistral) (Table S1), while the age model of core 479G is based on a combination of AMS ^14^C datings on planktic foraminifera (the date on benthic foraminifera was not included in the age model) and correlation to the NGRIP ice core and Heinrich events^32^.

According to the ^14^C datings (Fig. 2; Table S1), core 455G covers the period from the very final part of Marine Isotope Stage (MIS) 3 to the last millennium, ca. 30–0 kyr BP. High average sedimentation rates prevailed during late glacial conditions, but near 17 kyr BP sedimentation rates fell drastically. The age model of core 479G is based on a combination of AMS ^14^C datings performed on planktic foraminiferal tests and correlation to the NGRIP ice core and Heinrich events^32^; core 479G covers the interval from ca. 21–60 kyr BP (MIS 2–MIS 4), but only the upper part is shown here (Fig. 2).

### Sediment and ice-rafted debris events

**Figure 3:**
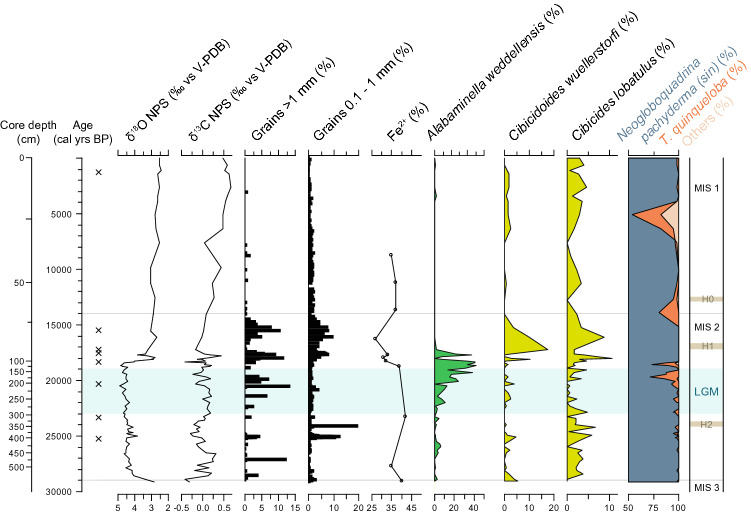
Selected data from core 445G: Stable oxygen and carbon isotope values on the planktic foraminifera *Neogloboquadrina pachyderma* (sinistral) (in ‰ vs. V-PDB), sediment grains > 1 mm and 0.1–1.0 mm and > 1 mm (in % of total sample) obtained through wet sieving, % of Fe^2+^ as well as the % of the high-productivity species *Alabaminella weddellensis*, the oxygen-demanding species *Cibicidoides wuellerstorfi* and the high-energy indicator *Cibidides lobatulus* (all calculated based on the total calcareous benthic foraminiferal assemblage). The cumulative relative frequencies of the polar planktic foraminifera *Neogloboquadrina pachyderma* (sinistral), the frontal indicator *Turborotalita quinqueloba* and other planktic species are calculated from the total planktic foraminiferal fauna (note that the scales starts at 50%). Marine Isotope Stages (MIS) are according to Lisiecki and Raymo^93^ and the Last Glacial Maximum (LGM; interval shown in blue-green colour) is placed according to Mix et al*.*^1^. “x” designated the location of ^14^C dates. The MIS 3/2 and MIS 2/1 boundaries are marked as gray horizontal lines, while the expected location of Heinrich (H) events are marked in light brown.

The sediment of 455G (Fig. S2) primarily consist of grey to olive-grey clay and silty clay with small angular gravel and stones. A 10-cm thick interval of lighter sediments (393–403 cm core depth; Figs. 2, 3) typical for intervals with detrital carbonate is seen near the base of core Sect. 7. Based on the ^14^C age model this lighter layer may be correlated to Heinrich (H) event 2 and the relatively high amounts of sediment grains > 0.1 mm surrounding this interval (Fig. 3), may be linked to iceberg discharge linked to the Heinrich event. Neither H1 nor H0 are visible to the naked eye. Intervals of a relatively high amount of sand (Fig. 3), i.e. IRD, surround the time period where H1 should be located, but the period represented by these larger grain sizes is too long to be linked to H1 with any certainty. Thus, neither H1 (ca. 24 kyr BP) nor H0 (ca. 16.8 kyr BP) are clearly expressed in the lithological record. However, temporally both H1 and H0 would fall within a section of the core, where accumulation rates are low and where the sediment is generally more coarse-grained, making the distinction of these Heinrich events difficult.

Core 479G sediments is dominated by olive-green to olive-grey clay with silt and fine sand interrupted by layers of sand and gravel^32^. Here, although H3 is shown as a distinct carbonate event, H2 is primarily identified through a minor peak in ice-rafted debris, while it lacks the light colour as seen in 455G, indicating that an increased carbonate content is only seen at the Labrador Sea site (455G), not in the Davis Strait (479G). Such a local difference in IRD provenance in this region has also been reported by Andrews et al*.*^34^.

Mössbauer spectroscopy data shows that the proportion of Fe^2+^ to the total Fe^2+^ and Fe^3+^ content in core 455G is 27–42% (Fig. 3, Table S2), with %Fe^2+^ displaying a marked minimum during the interval 18.5–~ 15.0 kyr BP, i.e. Fe^3+^ increase. Otherwise only relatively small variations can be observed. The results from X-ray diffraction measurements of core 455G samples show that the clay fraction is dominated by vermiculite in a mixture with smectite (Table S2), while smectite dominates at ~ 16 kyr BP (0.70 cm core depth), i.e. in the interval with Fe oxidation. Concurrently the clay content decreased (Table S2) and the sand fraction increased (Fig. 3). An increase in Fe^3+^ vesus Fe^2+^ represents increased oxidation of iron present in the clay mineral fraction (cf.^35^), which is in accordance with clay mineralogy data, showing that the clay mineral vermiculite is oxidized to smectite in this interval (Table S2). Thus the %Fe^2+^ may be used as a proxy for bottom-water oxygenation.

### Surface-water conditions: planktic foraminifera and stable isotopes

**Figure 4:**
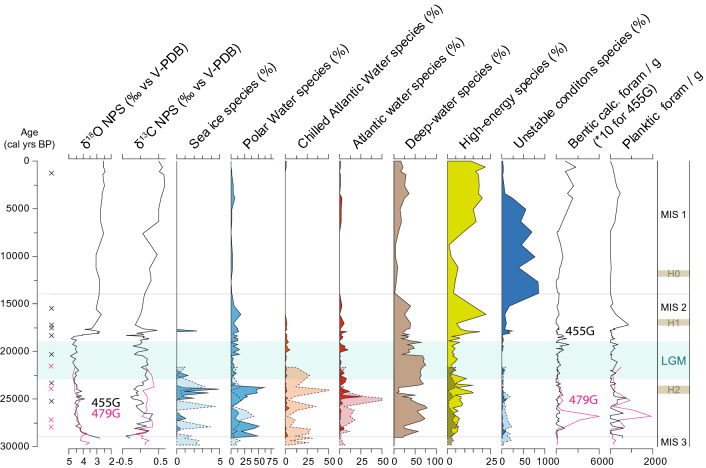
Stable isotope and foraminiferal distribution from cores TTR13-AT-455G (solid lines, dark colour) and TTR13-AT-479G (hatched lines, lighter colour). Benthic foraminifera are grouped according to environmental preference; calculated as percentage of the total benthic calcareous foraminiferal community. Sea-ice species: *Islandiella helenae*, *Stainforthia feylin*g*i*; Polar water species: *Cassidulina reniforme,* it may also tolerate highly chilled Atlantic water. Chilled Atlantic water: *Islandiella norcrossi*; Atlantic water species: *Cassidulina neoteretis,* Miliolida. Deep-water species (today found in NEADW): *Astrononion echolsi, Epistominella exigua, Ioanella tumidula, Melonis pompilioides, Nuttallides umbonifera, Oridorsalis tenerus, Pullenia bulloides, Pullenia subcarinata, Pullenia simplex, Tosaia hanzawai*. High-energy species: *Cibicides lobatulus, Discorbinella* spp. (mainly *Discorbinella araucana*), *Trifarina angulosa, Astrononion gallowayi, Astrononion stelligerum.* Species that tolerates unstable conditions: *Elphidium clavatum*. Note that *Cibicidoides wuellerstorfi* is not included here but shown separately in Fig. 3. Planktic stable isotope values (in ‰ vs V-PDB) were measured on left-coiled specimens of *Neogloboquadrina pachyderma*, while benthic and planktic foraminiferal concentrations are reported as number of specimens per gram wet sediment. Marine Isotope Stages (MIS) are according to Lisiecki and Raymo^93^ and the Last Glacial Maximum (LGM) is placed according to Mix et al*.*^1^. “x” designated the location of ^14^C dates for 455G (black) and 479G (purple). The MIS 3/2 and MIS 2/1 boundaries are marked as gray horizontal lines, while the expected location of Heinrich events are marked in light brown.

In both cores 455G (Fig. 3) and 479G, the polar species^36^ *Neogloboquadrina pachyderma* (sin.) dominates the planktic foraminiferal assemblages during the entire record, indicating cold polar surface water and likely extensive sea-ice conditions. There is a slight decrease in this polar species from 25 kyr BP onwards, i.e. also during the LGM, which, in concert with a peak in sea-ice related benthic foraminifera (ca. 25–23.5 kyr BP; Fig. 4), suggests periodically more open sea-ice conditions in connection with a potential partly breakup or thinning of previously (semi)perennial sea ice. However, only in a short interval at the termination of the LGM (in core 455G) the frontal and higher-productivity indicator *Turborotalita quinqueloba* makes up to 30% of the assemblage, suggesting warmer surface-water conditions and retreat of the sea ice (Fig. 3). This is also supported by the benthic foraminifera in core 455G, which during the end of the LGM and onset of the deglacial period, was characterised by influx of high-productivity indicators (Figs. 3, S3).

In both cores, the planktic foraminiferal δ^18^O and δ^13^C records are overall stable during the MIS 2 and LGM periods (Fig. 3, 4). This indicates overall stable surface water conditions, as supported by the very stable δ^13^C records inferring only limited changes in surface water mass and primary productivity. Notably there is a minor decrease in δ^18^O at ca. 25–23.5 kyr BP in core 455G, where also benthic sea-ice species increase, suggesting a minor decrease in surface-water salinity (Fig. 4; core 479G has too low a sample resolution to reliably identify this signal). Temporally this interval corresponds to H2, which may explain the lower surface-water salinities and disturbance in sea ice cover.

Shortly after the end of the LGM, the planktic δ^18^O record in 455G shows an abrupt shift to lighter values, most likely linked to increased glacial melting from the nearby Greenland and Laurentide ice sheets, possibly combined with warmer surface water conditions. Concurrently, the surface waters became gradually more enriched in ^13^C, presumably reflecting increased primary productivity using a significant amount of the ^12^C.

### Subsurface water (WGC base) conditions: benthic foraminiferal assemblages in 479G

Both today and during the LGM with its 120–140 m lower global sea level^37^, the benthic foraminiferal assemblage in core 479G (present water depth 1033 m) reflects oceanic conditions of subsurface water masses of the outer shelf to upper slope in the southernmost part of the Davis Strait. Core 479G itself does not encompass modern or Holocene deposits. However, at other sites along the West Greenland shelf from water depth corresponding to the present location of the core of the WGIW, the benthic foraminiferal assemblages today and during the Holocene are characterised by diverse faunas^38–40^, largely linked to the presence of chilled Atlantic Water (i.e., *I. norcrossi*) with comparatively low frequencies of the warmer, true Atlantic Water species^39–41^ (for details on species groupings see Methods and Figs. 3 and 4). In contrast, in 479G the benthic foraminiferal assemblage during MIS 2 was characterized by an overall high frequency of warmer Atlantic water indicator species (i.e., *C. neoteretis*) (Fig. 4, hatched lines). Thus, the foraminiferal assemblages of MIS 2 indicate that bottom-water conditions off West Greenland were saline and stable, with a similar, or possibly, with an even more prominent Atlantic Water signal than seen in the present subsurface WGIW of the WGC in the region. Radiocarbon measurements on benthic and planktic specimens at ~ 27,000 years ago show slightly younger ages for the benthic foraminifera (Seidenkrantz et al., 2019; Fig. 2) indicating that bottom-water conditions were well-ventilated within the entire depth range of the WGC, as previously also shown for the Holocene^40^.

Shortly prior to the LGM, the number of these warmer Atlantic Water indicators decreased compared to earlier in MIS 2, becoming replaced with species indicating more chilled Atlantic Water, inferring a continued Atlantic Water influx, but with increased mixing of these waters with colder Polar Waters. In accordance, the benthic foraminiferal assemblage is also influenced by species that thrive in Polar Water conditions. Thus, this mixture of cold- and warmer-water indicator species, suggests influence of both Atlantic and Polar water masses and variable hydrographic conditions of the subsurface water masses, as presently found off West Greenland and it may indicate a weaker transport or thinning of the WGIW mass and a gradual shift towards the Transitional Water that sweeps the site today (Fig. S1), even though present-day conditions were not reached until the Mid or Late Holocene (Fig. 4). Sinking of dense winter water or sea ice-induced brine formation may have played a role here. As far as our data are available, the influx of warmer, Atlantic-sourced subsurface water persisted during MIS 2 until at least 21.5 kyr BP (core top). This is notably also reflected in widespread high primary productivity.

### Deep-water (DWBU) conditions: benthic foraminiferal assemblages in 455G

With a present water depth of 2381 m, the 455G core site in the northernmost Labrador Sea represents intermediate to deep-water conditions, today swept by the NEADW. The benthic foraminiferal assemblage is dominated by typical deep-water species (Fig. 4, data shown as solid lines, Fig. S3), which today are found in areas characterised by NEADW^38^. However, particularly notable is the persistent presence of Atlantic Water indicator taxa during the LGM (Fig. 4). At deep to intermediate water depths, these taxa are today found in regions of the Labrador Sea influenced by upper NEADW or lower LSW, albeit in much lower frequencies^38^. This suggests that Atlantic-water influence at depth was stronger during MIS 2 than today and it was particularly strong after ca. 25 kyr BP. Apart from an interval in the mid LGM (ca. 22–21 kyr BP) this regime persisted, with some variability, through the LGM until ca. 15 kyr BP, albeit weakening in the later millennia. In contrast, most of the pre-LGM section of MIS 2, was, apart from the more ubiquitous NEADW deep-water species (Fig. 4), to a larger extent dominated by Polar Water and sea ice species, although with several shifts in dominance of warmer and colder-water species (Fig. 4). This indicates that during MIS 2, ocean circulation was variable, but the influence of intermediate warm Atlantic-sourced water was generally high. The end of the LGM and onset of the deglacial period was characterised by a high influx of high-productivity indicators (*Alabaminella weddellensis*; Figs. 3, S3), suggesting high food availability during local breakup of sea ice, as indicated by the presence of *T. quinqueloba* (Fig. 3).

The following period from ca. 18.5 to ~ 15 ka was characterised by unusually high percentages of *Cibicidoides wuellerstorfi* (Fig. 3), a species requiring high bottom-water oxygenation. The concurrent marked decrease in the percentage of Fe^2+^, which originated from an increased oxidation of iron present in the clay mineral fraction^35^, supports an increased oxygenation of the surface sediments (Fig. 3). These data thus suggest a distinct, but relatively short-term episode of increased ventilation of bottom-water lasting for a few millennia.

The deglacial period and much of the Holocene is dominated by species suggesting unstable bottom water conditions or possible down-slope transport of sediments in a high-energy environment, while some intervals with high-productivity species (Fig. S3) suggest increased vertical mixing of upper water masses.

## Discussion

The planktic foraminiferal records from both cores yield information on circulation changes of the upper waters of the (proto) WGC system, whereas the benthic records from the two cores manifest circulation changes in both the (proto) WGC subsurface waters and the deeper (NEADW stratum) waters, respectively. Thus, the benthic record of core 479G reflects circulation changes at the transition between WGC-entrained WGIW and the underlying TrW, while the benthic record of core 455G is representative of changes in deeper water circulation of the DWBU, i.e. the NEADW, and the overlaying LSW. This combination of proxies and cores from different water depths in the boundary current zone off SW Greenland allows us to reconstruct MIS 2 circulation of the NE Labrador Sea—southern Davis Strait for the upper ca. 2250 m of the glacial water column.

### Persistent (WGC) subsurface and intermediate-deep (DWBU) Atlantic water influx to the Labrador Sea

**Figure 5:**
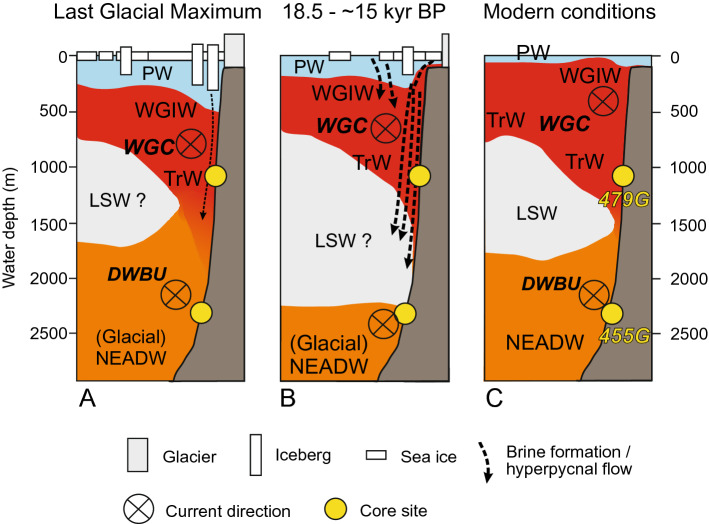
Schematics of interpretation of water mass distribution near our core sites off the shelf of West Greenland: (**A**) Last Glacial Maximum, (**B**) ca. 18.5–15 kyr BP, and (**C**) Modern. The modern conditions are interpreted from Hall et al.^94^ and the World Ocean Atlas^92^. PW, Polar Water; WGIW, West Greenland Irminger Water; TrW, Transitional Water; NEADW, North East Atlantic Deep Water; LSW, Labrador Sea Water; WGC, West Greenland Current; DWBU, Deep Western Boundary Undercurrent.

While surface-water conditions were cold with extensive sea ice during the LGM (Fig. 5), the entire period of MIS 2 was characterized by the influx of saline and relatively warm Atlantic-sourced water, which had a stronger signal than seen in the area today^38, 42^, both at subsurface (479G) and greater (455G) depths (Fig. 4). The influx of Atlantic-sourced water as well as associated bottom-water transport activity at subsurface levels (479G) may have been slightly weaker during the LGM itself than the pre-LGM period of MIS 2. The strong Atlantic-sourced signal could in part be explained by a weaker Polar water flow from the East Greenland Current, but the Atlantic water signal appears so prominent that this cannot be the only explanation. Presently, Atlantic water can be found at subsurface depths below ice tongues and sea ice in northern Greenland^43, 44^. A thick sea ice cover, reducing the melting from the Greenland ice sheet and thus the mixing of Atlantic Water with cold Polar Water during glacial conditions, could potentially cause the subsurface Atlantic Water to retain a higher temperature and salinity. Nevertheless, the temperature and velocity of the WGIW is today to a large extent governed by the North Atlantic boundary current conditions^25^, which may also apply to MIS 2. The present-day advection of WGIW is at maximum strength during winter, when the Irminger Current is at a maximum^25^. We suggest that this may at least in part explain the strong subsurface Atlantic Water signal during glacial times, when a cold Northern Hemisphere climate with extensive continental ice sheets may have resulted in year-round strong temperature and atmospheric pressure gradients, somewhat comparable to present-day winters. At the same time none of the deep-water and Atlantic water indicators in the benthic foraminiferal fauna shows evidence of reduced bottom-water oxygenation during MIS 2, suggesting a persistent inflow of NEADW.

Hence, pre-LGM strong convection in parts of the northern North Atlantic may have involved both deep (e.g., NEADW) and subsurface water (e.g., Irminger Sea Water) masses (Fig. 4). It may be linked to larger-scale AMOC intensification in the North Atlantic just prior to the LGM as also reflected by an episode of stronger overflow through the Faroe-Shetland Channel at the end of MIS 3^45^. At the deep/intermediate water level at 455G, Atlantic-sourced water influx was present from before the LGM until ca. 15 kyr BP.

Many studies have shown very cold, lower-salinity surface waters^46^ and an extensive sea ice cover in the Labrador Sea during the LGM^2, 47^. It has been suggested that these cold surface waters directly overlay the deep and bottom water masses of the basin^48^, suggesting reduced or absent influx of Atlantic (sub)surface water. However, later studies indicate that Atlantic-sourced subpolar water have penetrated northwards into the Baffin Bay already since the early deglaciation^49, 50^. Today warmer Atlantic waters penetrate deep into many Greenland fjords underlying polar surface water masses^43^. In analogue, warmer and saline waters of the North Atlantic Current reach far north into the Nordic Seas and the Arctic Ocean. This actual scenario also applies to subsurface circulation in the Nordic Seas during glacial Heinrich events^51^. Based on the warm subsurface waters recorded in core 479G, we suggest that also during the LGM, warm and saline subsurface waters were transported northward by the North Atlantic (Irminger) Current and subsequently advected into the Labrador Sea through boundary current transport, while underlying the cold and fresher surface waters (Fig. 5). This provides evidence for a relatively strong Irminger Current (cf.^25^) and thus also likely a stronger SPG and AMOC than previously assumed^52^, which together with an extensive sea-ice cover allowed an effective transfer of saline (subsurface) water masses from lower latitudes into the (sub)polar region.

The benthic foraminiferal fauna in core 455G suggests strong but variable DWBU conditions and an active AMOC at subsurface and intermediate water depths during the LGM, transporting upper NEADW into the Labrador Sea. Some of the variability of the foraminiferal assemblages may potentially be ascribed to varying conditions in the far-field source regions for the Atlantic water, as previously suggested for the deglacial period^38^. However, the latter reported variations^38^ were primarily linked to bottom-water oxygenation and our data show no indication of possible changes in source region during the glacial period (Fig. 4), which suggests that it is indeed transport strength which has been a crucial factor for the changes in water mass composition recorded in our study region. Early studies indicated that convection in the North Atlantic was significantly limited during the LGM^13, 53^ and that the Atlantic Meridional Overturning Circulation (AMOC) was slower than today^54, 55^. It has been suggested that a decreased northward transport of heat and salt by the North Atlantic Current^56^, combined with reduced evaporation related to tropical cooling^57^, resulted in a decreased salinity of the North Atlantic^58^. As discussed previously, the convection in the North Atlantic was believed to primarily take place south of Iceland with the deeper waters only reaching intermediate water depths (GNAIW^16^). However, later studies suggested that production of North Atlantic Deep Water (NADW), including GNAIW, took place in the North Atlantic also under glacial conditions^4, 59^. Yet, these authors could only speculate about the location of these possible sinking sites for these northern-sourced deep-water masses^4^, while recently is was proposed that some LGM deep-water formation occurred in the Labrador Sea^14^. Evidence for active formation of NADW during the LGM has recently also been provided by Klockmann et al*.*^60^, while Ezat et al*.*^61^ suggest the possibility of enhanced Arctic Ocean convection during the LGM. Also Pöppelmeier et al*.*^62^ support a northern source of both intermediate and deep water during the LGM, suggesting that the distribution of water masses in the Atlantic was not much different than today.

Presently, the SPG plays an important role for the distribution of heat and salt in the North Atlantic region^20, 63, 64^ and it provides the mechanism for introducing saline, Atlantic-derived water masses at both subsurface (WGIW via the WGC) and deeper levels (NEADW by the DWBU) to the Labrador Sea. Together with surface water cooling, the elevated salinities of the Atlantic water provide the mechanism for destabilizing the water column and initiating deep convection, i.e. Labrador Sea Water formation^65^. Our finding of inflow of both subsurface (WGC) and intermediate Atlantic (NEADW) waters into the Labrador Sea suggests a persistent boundary current activity, which may have been linked to an active SPG during most of MIS2. A strong SPG may also have played a role for LGM deep convection^66^, as deep mixing processes and open ocean deep convection is particularly favoured under conditions of a strong SPG circulation regime. Prevalence of wind patterns favouring such a LGM regime is indicated by LGM atmospheric modelling results^67^. Together with reduced LGM precipitation over the western SPG^68^, strong SPG cyclonic circulation has been found to lead to eddy salt fluxes promoting formation of dense waters and deep mixing in the SPG centre, while a weak circulation results in freshening which prevents convection^46^. A strong influx of Atlantic-derived (WGIW) subsurface water masses may also have played a crucial role in the very early (19–18 kyr BP) deglaciation of the Southwest Greenland shelf^69^.

### Reduced NEADW inflow during Heinrich event 2

Heinrich event 2 can be recognised as a distinct interval in the lithological profile (Fig. S2). It also stands out in the planktic oxygen isotope and benthic foraminiferal records (Figs. 3, 4), especially in the higher-resolution core 455G. Here planktic δ^18^O was depleted (Fig. 4), suggesting reduced surface water salinities, in accordance with increased frequencies of benthic foraminiferal sea-ice and polar water species. At 479G, Atlantic Water species still persisted but gradually became less dominant, being substituted by species indicating Chilled Atlantic Water. In contrast, at the deep site 455G the Atlantic Water species thrived while the deep-water species almost disappeared during H2. We suggest that the release of icebergs during H2 resulted in sea-ice expansion and a thick surface to subsurface layer of lower-salinity Polar Water which was mixed with glacial meltwater. The PW likely pressed the inflowing Atlantic Water to deeper levels of the water column, where it would have mixed with the deeper waters. At the same time, the flow of NEADW may temporarily have decreased in accordance with a weakened AMOC and NADW formation^70, 71^. This may also have involved an enhanced subsurface and intermediate water flow as previously suggested to have occurred prior to or during Heinrich events (e.g.,^70, 72, 73^). The deepening of the Atlantic Water strata caused a gradual warming at depth, first affecting subsurface waters and later also the deep waters in our study area. The resulting destabilization of the benthic environment may have led to a near-collapse of the deep-water benthic foraminiferal community during this period (Fig. 4).

### Brine formation and Labrador Sea Water formation during the deglacial phase

At the end of the LGM, ca. 20–17 kyr BP, a significant bloom in the foraminiferal species *Alabaminella weddellensis* (Fig. 3) indicates a period of high primary productivity (cf.^74^). We suggest that this productivity interval was linked to breakup of perennial shelf sea ice, as also indicated by warmer surface waters inferred from the planktic foraminiferal record (Fig. 3). Support for enhanced advection of warm and saline WGIW in the time interval close to 17 kyr BP was presented by Knutz et al*.*^75^, who reported abnormally high alkenone-based surface water temperatures for this period. Following this period of less extensive sea ice and warmer surface water, the planktic foraminiferal fauna indicates that surface waters again cooled, when planktic oxygen isotope ratios indicate an abrupt shift to lighter values indicating the onset of melting of the surrounding ice sheets (Fig. 3). Concurrently, the Fe^2+^ record (Fig. 3) and a peak in the epifaunal species *Cibicidoides wuellerstorfi* both indicate a period of strong bottom-water oxygenation^35, 76^, while a peak in sand content (Fig. 3) and overall low foraminiferal concentrations (Fig. 4) suggest increased sediment transport activity and current speed at the site. Yet, despite the increased sand content there is no further significant change in sediment composition, nor is there any indication of downslope transport of foraminifera during this interval, suggesting only limited downslope transport of sediments from shallower sites. Some of the coarser sediment may have reached the site as ice-rafted debris, but this would not explain the oxygenated bottom waters. Based on the Fe^2+^ alone, we cannot rule out that additional oxygenation events had occurred earlier in the record, and certainly today a significant variability is seen in the LSW oxygenation^64^. However, the foraminiferal data show no evidence of such previous bottom-water oxygenation events, suggesting that such deep ventilation events were confined to parts of the deglaciation period. The timing of the oxygenation episode corresponds to a period surrounding Heinrich event 1, but based on the available data we cannot conclude if the cold H1 environment may have played some role in the oxygenation processes. Within this context it must be noted that the oxygenation episode had started significantly earlier than H1.

The location of core 455G at the mouth of a canyon originating from the Greenland shelf further north (Fig. 1B) may have made it particularly sensitive for input of hyperpycnal sediment-laden meltwater masses^77^ and/or dense brine water masses released during sea-ice formation upslope^26^. Keigwin and Swift^14^ suggested that glacial deep-water formation processes in the North Atlantic presumably were related to brine rejection associated with sea ice formation along the Labrador Sea margin. Also today there are indications of formation of dense winter water on the Greenland shelf and subsequent down-slope flow of these water masses into the deep Labrador Sea basin^26, 78^, and similar processes may thus also have occurred under post-glacial conditions. Hence, we here suggest that the extensive bottom-water oxygenation was linked to a period of downslope hyperpycnal flows and brine water transport (Fig. 5), potentially leading to LSW formation (Fig. 5). These processes would be linked to large-scale melt-water release and sea ice formation over the SW Greenland shelf triggered by increased Greenland Ice sheet melting and iceberg drift during the early deglaciation^68^. A further factor promoting deep convection and extra supply of oxygen to the deep Labrador Sea may have been severe winter-time cooling^64^ associated with a generally cold North Atlantic climate regime at the time of H1. Within this context, a recent study of glacial-interglacial variability of North Atlantic Deep Water notably found that the deep western Atlantic was as well-ventilated during Heinrich Stadial 1 as during the LGM^59^.

The age of the Fe^2+^ minimum interval (Fig. 3) corresponds to the timing of similar geochemical changes reported from the Faroe overflow region, which previously have been interpreted as a more local phenomenon^35^. Both our present study site and the Faroese site are located along the North Atlantic transport pathway of water masses originating from (sub)polar deep-convection processes, in this case presumably in relation to brine formation. A similar timing of enhanced oxidation of sediments at these distant sites could indicate that a generally enhanced SPG subsurface or intermediate water circulation also occurred, in analogue to what recently was observed in the 1990’s^19^. It is thus possible that an episode of LSW formation triggered by local brine formation may have been sufficiently strong to transport oxygenated water at intermediate levels to a larger section of the northern North Atlantic.

The episode of extensive bottom-water ventilation was terminated just prior to ~ 15 kyr BP, when the deep warmer-water influx into the Labrador Sea ceased and bottom water conditions became more unstable. This may have involved, among other processes, an inflow of relatively oxygen-poor Atlantic-derived water masses at depth and the establishment of more variable deep-water conditions, which prevailed until early Holocene times^38^. The timing of this change close to 15 kyr BP is coeval with the initiation of a more persistent strong overflow of the Greenland–Scotland Ridge^18^. This persistent overflow likely resulted in density changes in the deeper parts of the Labrador Sea basin, which may have caused changes in the vertical current speed profile in the boundary current zone along the Greenland slope. The drastic decrease in sedimentation rate in core 455G (Fig. 2) during the deglaciation may reflect such a change in the general deep-water current and sedimentation regime, while abundant IRD in a record retrieved close to the 455G site suggest that a comparably ‘modern type’ boundary current circulation may have started already during early deglacial times^75^. An early re-initiation of the DWBU shortly after the LGM has previously also been reported by Fagel et al.^79^, who stated that current activity gradually increased and reached its maximum in the Holocene. More generally, it should be noted that under an active pre-Holocene SPG regime sedimentation conditions were significantly different than later in the Holocene. Widespread input of sediments transported through melt water from a proximal, and subsequently retreating, ice front on the shelf favoured high sedimentation rates on the slope^64^. After the postglacial retreat of the ice margin melt water sediment transport significantly declined and contour current activity became prevalent^26^).

## Conclusions

Based on foraminiferal, sediment and geochemical records of two marine sediment cores from the northern Labrador Sea (2281 m water depth) and the Davis Strait (1033 m), we found new evidence of strong boundary current activity and enhanced ocean convection during the severe glacial conditions of the LGM. Our study shows that below the cold polar surface waters, extensive influx of Atlantic-sourced water characterised both subsurface and intermediate to deep water conditions in the northeastern Labrador Sea. At subsurface levels the Atlantic-sourced waters were likely carried by a strong (proto) West Greenland Current, while the deeper water mass had characteristics comparable to present upper Northeast Atlantic Deep Water (Fig. 5). Despite some variability, this scenario with a strong Labrador Sea boundary current was overall stable for most of Marine Isotope Stage 2, including the LGM. It was however temporarily interrupted during Heinrich event 2, where our record suggest a weakened NEADW and a deepening of the Atlantic Water subsurface layer.

The first indication of a prominent, glacial oceanographic change occurred at the initial surface-water warming, and likely breakup of sea ice, at ca 20 kyr BP. A significant shift in planktic stable oxygen isotopes at ca. 19–18 kyr BP, was coeval with the onset of a decrease in Fe^2+^ and an increase in oxygen-demanding foraminifera, indicating a period of significant bottom-water oxygenation from 18.5 to ~ 15 kyr BP. The isotopic shift to depleted values likely marks the initiation of significant melting of the Greenland Ice Sheet^68^, which may have caused hyperpycnal flows of sediment-laden melt water, transporting oxygen-rich waters to the deep basins, while the resulting cooling of surface waters may have triggered sea ice and brine formation (Fig. 5). Both processes may have led to enhanced Labrador Sea convection during the early deglaciation. A major change and re-arrangement of deep-water masses and current conditions in the deep Labrador Sea basin at ca. 15 kyr BP may be related to the concurrent initiation of a stronger, persisting overflow of the Greenland-Scotland Ridge suggested previously^18^.

This is the first data-based study that presents indications for the existence of a strong glacial Subpolar Gyre with an active Labrador Sea boundary current regime at both subsurface and intermediate/deep levels during the LGM. A strong Meridional Overturning Circulation cell in the North Atlantic during the LGM has previously been proposed by models^4^, but here we show the first data to support this hypothesis. The strong influx of Atlantic-derived subsurface waters may have played a role in the very early (19–18 kyr BP) deglaciation of the Southwest Greenland shelf^68^.

## Methods

Eight ^14^C datings were performed for chronostratigraphic control of core 455G at the Aarhus AMS ^14^C Dating Centre, Aarhus University (Table S1), using the planktic foraminifera *Neogloboquadrina pachyderma* (left-coiled). We used the depositional model option in the OxCal 4.2 software^80^ and the Marine13 calibration curve^81^ and ΔR = 140 ± 35 years in keeping with previous studies from the West Greenland region^32, 48^ to establish the age model (Fig. 2). In the North Atlantic region significantly larger reservoir ages have been suggested for the last glacial period^82, 83^, and this may also potentially have been the case for off West Greenland. However, recently Ezat et al*.*^61^ showed that during the LGM, the North Atlantic was not filled with very old bottom waters during the LGM, as previously suggested. Also, in core 479G, ^14^C was measured both on benthic and on planktic foraminiferal specimens in one sample from the MIS 2 interval^32^. Here the benthic foraminiferal sample suggests a slightly younger age than a date based on planktic foraminifera from the same depth, indicating strong ventilation of the water column also during glacial times^32^. This would suggest that the glacial reservoir age might in fact not have been very different than present day values at our study site. Notably, even with a potentially higher-than-present reservoir effect during glacial, our chronology clearly shows high-resolution records of MIS 2 in both cores.

The age-depth model of Core 479G, is described in detail in^32^. According to this age model, core 479G covers the interval from ca. 21–60 kyr BP (MIS 2–MIS 4), but here we focus on the MIS 2 interval (Fig. 2).

Information on the grain size distribution of samples representing 1-cm sediment slices were carried out through wet-sieving of the sediment through sieves with mesh sizes of 0.063, 0.1 and 1.0 mm. The sediment grain-size fractions 1.0–0.1 mm and > 1.0 mm fractions (in % of the total wet sediment sample) in 513 samples from core 455G is shown in Fig. 3; the larger fractions may be considered to represent ice-rafted debris (IRD), while the 1.0–0.1 mm fraction may encompass both IRD and sediments deposited through high current speeds, although it should be noted that the 0.1–1.0 mm fraction also includes benthic and planktic foraminifera. Due to a high content of siliceous sponge spicules in the lower part of core 455G, the 0.01–0.0063 mm fraction could unfortunately not be calculated.

For benthic and planktic foraminiferal analyses, the non-dried samples were washed through sieves with mesh sizes of 0.063, 0.1 and 1.0 mm and foraminifera were counted from the 0.1–1.0 mm fraction. The 0.063 mm fraction of 50 selected samples was checked for additional species; only few foraminiferal specimens were present in this fine fractions, none of them other than those found in the 0.1–1.0 mm fraction. Where possible, we counted at least 300 benthic and 300 planktic individuals in each sample. However, due to low foraminiferal concentrations, this was not always possible and only samples with more than 40 benthic or planktic specimens were included in the respective calculations. Planktic foraminiferal assemblages were analysed in detail in core 455G, while the overall trend seen in 455G were only confirmed in 479G through a more cursory study. In 455G, analyses were originally carried out on 1-cm sediment slices for every 3–10 cm, but often low number of foraminiferal counts meant that a number of surrounding 1-cm slices has to be counted and data from several cm sediment had to be combined into one sample; consequently the 212 samples analysed had to be combined to a total of 66 benthic and 108 planktic foraminiferal samples reported (Figs. 3, 4, S3). However, the samples, where the number of foraminifera was too low to be included in the percentage calculations, are still included in the calculations of benthic and planktic foraminiferal concentrations (Fig. 4). The statistical error in foraminiferal assemblages depends on the number of specimens counted. Counts of 300 specimens in a sample is sufficient for detailed interpretations and species making out at least 3% of an assemblage may be considered to be statistically reliable^84^, while values < 1% may cease to be meaningful^85^, although the data are still useful as part of the general context. With lower counts, the statistical significance is reduced, but clear changes in assemblages are still reliable. We show 19 samples from 479G.

Benthic foraminiferal assemblages are among the most valuable proxies for estimating past bottom water conditions. The species distribution depends on temperature, salinity, stability, food availability and type, oxygen levels and to some extent, water depth, with some species more dependent on temperature and/or salinity while others on food availability. Due to the different physical parameters characterising the different water masses today (e.g.^27, 29^) and the ecological requirements of the foraminiferal species it is possible to develop a “foraminiferal fingerprint” of these water masses. Thus, it is possible to determine the characteristics of the dominant water mass at a given site in the past. As Arctic benthic foraminifera may grow primarily during the spring–summer-fall season^86^, our benthic foraminiferal records likely represents a “warm”-season environment. The ecological preferences of the species encountered in this study is relatively well known and our ecological interpretations are based on the modern habitat requirements of the species (e.g.,^38, 42, 44^). Although any given species is rarely dependent on only one parameter, they most commonly have a primary ecological requirement; here we have grouped the benthic foraminiferal species according to their primary habitat. Species groups show in Fig. 4 are as follows: Sea-ice species: *Islandiella helenae*, *Stainforthia feylin*g*i*; note that benthic foraminiferal sea-ice species are normally not found under perennial sea ice but in connection to sea-ice margins or open-water areas within the sea-ice cover^87^*.* Polar water species (medium blue): *Cassidulina reniforme*, may also tolerate highly chilled Atlantic water. Chilled Atlantic water species (i.e. Atlantic Water blended with Polar water) (orange): *Islandiella norcrossi.* Atlantic water species (red): *Cassidulina neoteretis*, Miliolida. Deep-water species (NEADW, brown): *Astrononion echolsi, Epistominella exigua, Ioanella tumidula, Melonis pompilioides, Nuttallides umbonifera, Oridorsalis tenerus, Pullenia bulloides, Pullenia subcarinata, Pullenia simplex, Tosaia hanzawai*. Species that are found in areas swept by Labrador Sea Water today include^38^ (LSW, not shown in a specific group): *Stainforthia concava*, *Pullenia subcarinata*, *Cassidulina neoteretis*, *Melonis barleeanus*, *Cibicidoides wuellerstorfi*, *Nuttallides umbonifera*, *Cibicides lobatulus*. High-energy species (yellow green)**:** *Cibicides lobatulus, Discorbinella* spp. (mainly *Discorbinella araucana*), *Trifarina angulosa, Astrononion gallowayi, Astrononion stelligerum.* Note that *Cibicidoides wuellerstorfi* is not included in the high-energy group of Fig. 4 and shown separately in Fig. 3. Unstable conditions (dark blue): *Elphidium clavatum*; it cannot be ruled out that this species is transported from shallower sites, although it is notable that no other species that would indicate down-slope transport are present. For further detail to species groupings, incl. references, see the Supplementary Material. A list of species and author names for taxa mentioned in text and figures given Table S3.

Oxygen and carbon isotope measurements of the planktic species *Neogloboquadrina pachyderma* (sinistral) were performed at Woods Hole Oceanographic Institution (WHOI) on a Finnigan MAT252 mass spectrometer with a standard deviation of the isotope values in accordance to the National Bureau Standards (NBS) carbonate standards NBS-19 and following the procedure described by Ostermann and Curry^88^. For core 455G, 76 samples were analysed, while 19 samples were measured for core 479G in the interval presented here. The specimens were picked from the 0.1–1.0 mm fraction, but care was taken to select specimens from the 0.15–0.25 mm fraction. All values are calibrated to the V-PDB scale.

The Mössbauer spectroscopy (Fig. 3, Table S2) was performed at GEUS, Copenhagen, using a constant acceleration spectrometer with a 50 mCi source of ^57^Co in Pd to detect the content of Fe^3+^ and Fe^2+^. Samples were analysed both at liquid nitrogen temperature and at room temperature. Spectra were computer fitted by Lorentzian line shapes. The samples analysed at room temperature were fitted with two doublets representing Fe^3+^ and Fe^2+^, while the spectra analysed at liquid nitrogen temperature were fitted with one sextet and two doublets. This is the same method as used by Lassen et al*.*^35^.

For the clay mineralogical analyses (GEUS, Copenhagen), the samples were treated according to the method presented by Lassen et al*.*^35^. Samples were dispersed by ultrasonic treatment and the fractions were subsequently isolated by centrifugation and each fraction was saturated with Mg^2+^ or K^+^. Samples saturated with Mg^2+^ were air-dry or treated with ethylenoglycol, while those saturated with K^+^ were heated to 300jC. They were afterwards investigated by Co-K a radiation on a X-ray diffractometer using a Philips 1050 goniometer with fixed 1/4^o^ divergence and scatter slits. The relative amounts of clay minerals were estimated semiquantitatively from the reflection areas in the diffractograms and the relationship between vermiculite and smectite is reported in Table S2.

For further details of core 479G analyses see^32^.

## Supplementary Information

Supplementary Information 1.

Supplementary Information 2.
