# Supplementary material for: Evidence for influx of Atlantic water masses to the Labrador Sea during the Last Glacial Maximum
Source: Sci Rep. 2021 Mar 24;11:6788. doi: 10.1038/s41598-021-86224-z (PMC7991648; doi:10.1038/s41598-021-86224-z)
Supplement: Supplementary file 1 — Supplementary Information 1. [file 41598_2021_86224_MOESM1_ESM.pdf]

Supplementary material to

Evidence for influx of Atlantic water masses to the Labrador Sea during the Last Glacial Maximum

Marit-Solveig Seidenkrantz, Antoon Kuijpers, Steffen Aagaard-Sørensen, Holger Lindgreen, Jesper Olsen, Christof Pearce

Content

The below supplementary material provide additional information on core TTR13-AT-455G. For similar information on core TTR13-AT-479G see Seidenkrantz et al. (2019) and supplementary material herein.

- Oceanographic transects from World Ocean Atlas
- Sediment description of core TTR13-AT-455G
- Chronology of core TTR13-AT-455G
- Mössbauer spectroscopy data
- Foraminiferal assemblage data and ecological groups
- Extended discussion: Antarctic Bottom Water
- References

Oceanographic transects from World Ocean Atlas

The present water mass distribution in the study area is illustrated through temperature and salinity transects across the two core sites: is located in the northernmost Labrador Sea (core site TTR-13-AT-455G and in the southernmost Davis Strait (core TTR-13-AT-479G) (Fig. S1). Water masses are indicated. PW = Polar Water from the Arctic ocean mixed with local Greenland meltwater; WGIW = West Greenland Irminger Water, i.e. warm Atlantic-sourced water; TrW = Transitional Water, a mixture of Atlantic and Polar water with still a relatively warm signal; LSW = Labrador Sea Water; NEADW = North East Atlantic Deep Water. Data are obtained from the World Ocean Atlas 2018 and plotted using the Ocean Data View program.

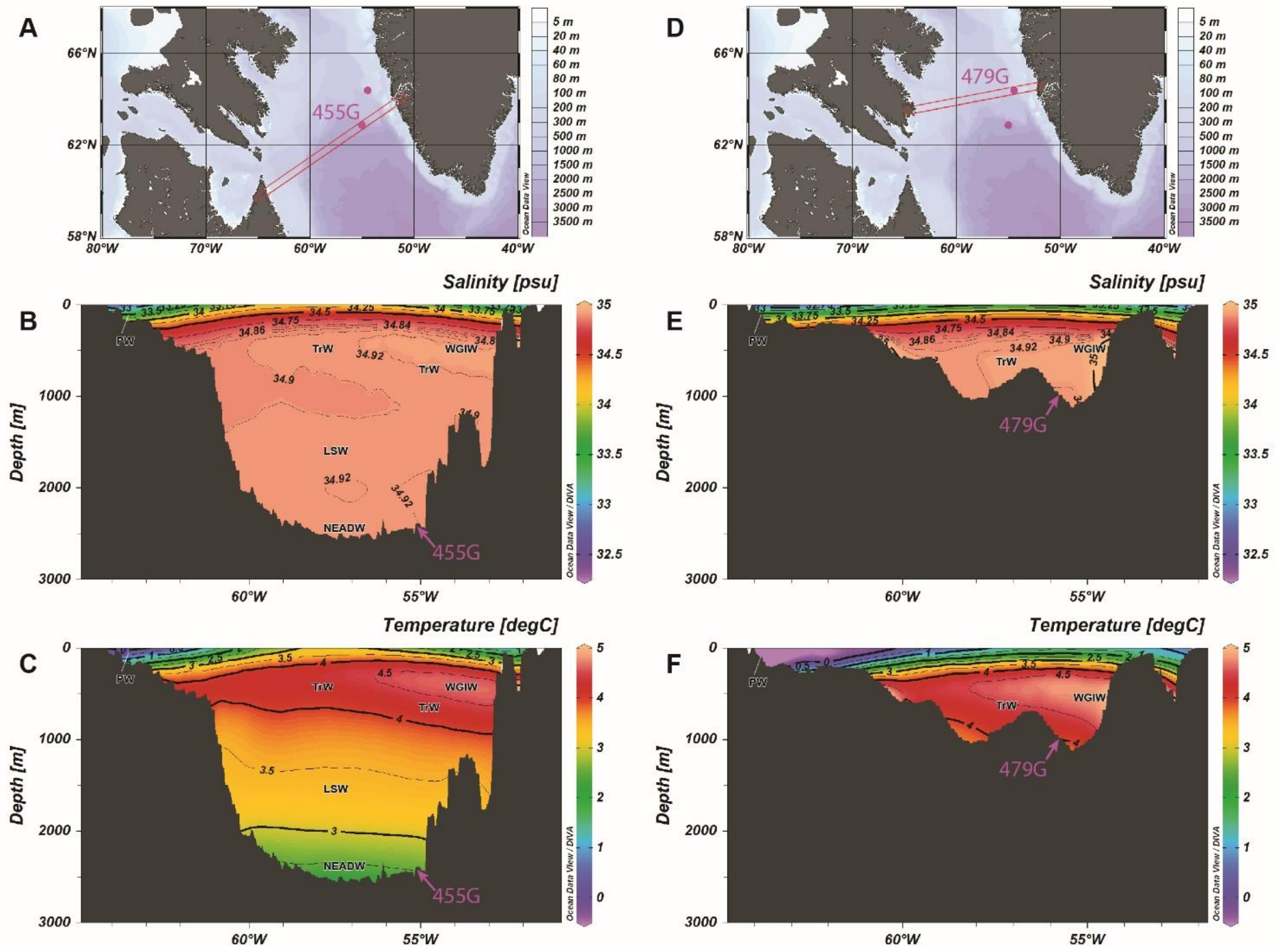

Figure S1. Temperature and salinity transect across our core sites extracted from the World Ocean Atlas 2018 (Locarnini et al., 2018; Zweng et al., 2018) and plotted using the program Ocean Data View, version 5.3.0 (https://odv.awi.de/). The location of the transects are shown in A) and D); for each transect data are derived from the area framed by the red lines; core sites are marked with purple dots. B) Salinity and C) temperature profiles across TTR13-AT-455G in the northernmost Labrador Sea. E) Salinity and F) temperature profiles across TTR13-AT-479G in the southern Davis Strait. Approximate location of water masses are marked on B, C, E and F. PW = Polar Water, WGIW = West Greenland Irminger Water, TrW = Transitional Water, LSW = Labrador Sea Water, NEADW = North East Atlantic Deep Water.

Sediment description of core TTR13-AT-455G

Gravity core TTR-13-AT-455G (hereafter 455G) was retrieved from the northernmost part of the Labrador Sea (62°52,17'N; 55°11,22'W; 2381 m water depth; Kenyon et al., 2004). The sediment (Fig. S2) primarily consist of grey to olive-grey clay and silty clay. One 10-cm thick interval of lighter sediments typical for intervals with detrital carbonate is seen near the base of core section 7. Based on the ^{14}C age model, this interval corresponds to Heinrich event 2 (see Hemming, 2004).

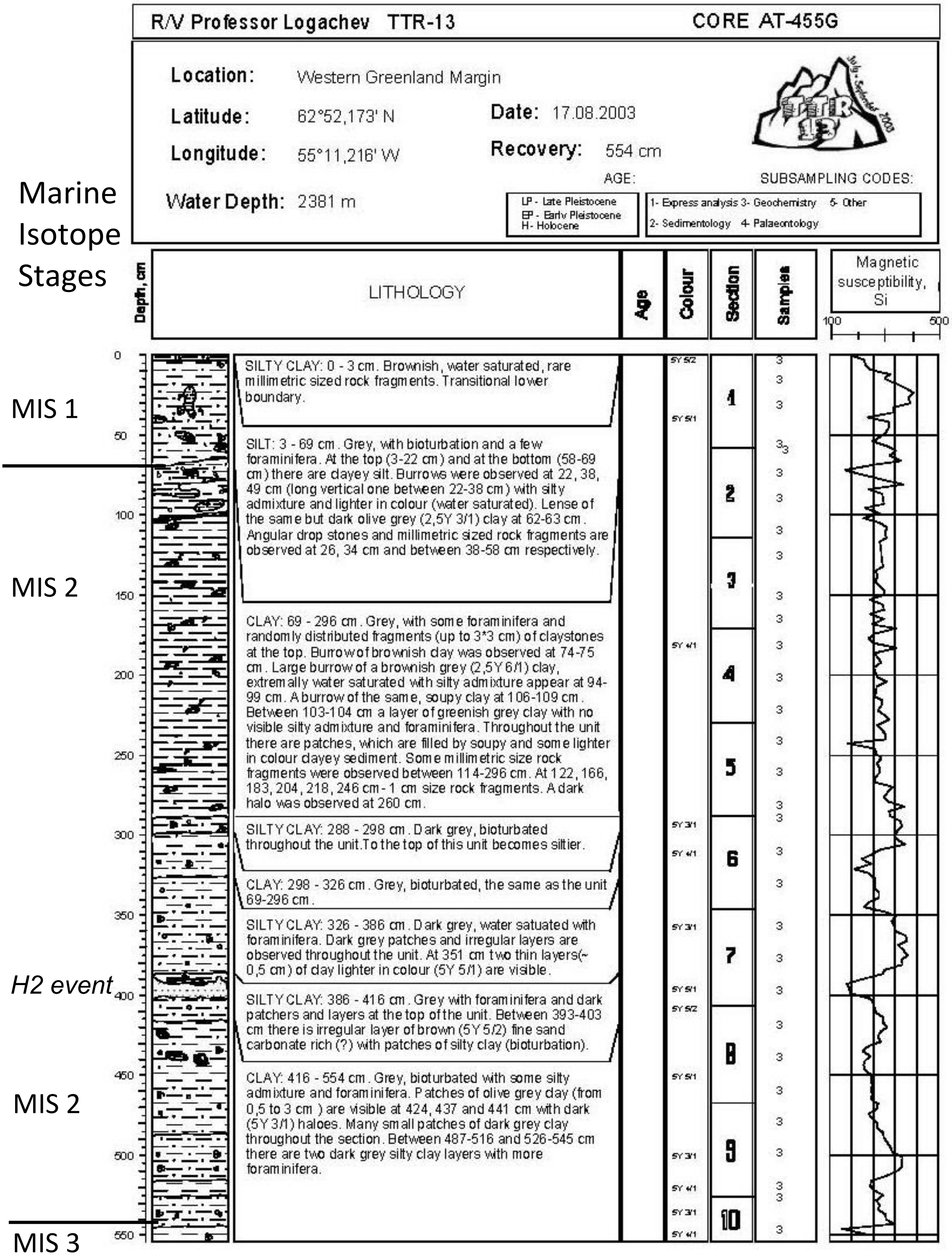

Figure S2. Sedimentary description carried out during the cruise (Kenyon et al., 2004). Marine Isotope Stages (MIS; Lisiecki & Raymo, 2005) and Heinrich (H) event 2 are marked (Hemming, 2004) based on the ^{14}C chronology (Fig. 2, Table S1). The Last Glacial Maximum corresponds approximately to the interval from 140-300 cm core depth. Sediment colours are 5Y 3/1 to 5Y 5/1 on the Munsell colour scale.

Chronology of core 455G

Eight AMS ^{14}C-datings were performed for core 455G at the Aarhus AMS ^{14}C Dating Centre (AARAMS), Aarhus University (AAR), (Table S1; Fig. 2), using the planktic foraminifera *Neogloboquadrina pachyderma* (sinistral, left-coiled). We used the depositional model option in the OxCal 4.2 software (Bronk Ramsey, 2009) and the Marine13 calibration curve (Reimer et al., 2013) with a ΔR = 140±30 years to establish the age model (Fig. 2) in accordance with previous studies from the West Greenland region (Lloyd et al., 2011; Sheldon et al., 2016; Jennings et al., 2017).

Table S1. Chronological information for core TTR 13 AT 455 G. Radiocarbon ages are calibrated using Marine13 (Reimer et al., 2013) with a ΔR of 140 ±35 14C years.

Lab.code	Sample name	Depth	Material	^{14}C age	Modelled age range (kyr BP)			δ^{13}C	δ^{18}O
		(cm)		(^{14}C yrs. BP)	68.2%	95.4%	μ ±σ	‰VPDB	‰VPDB
AAR-10060	TTR13AT 455G, 9-11 cm	9-11	*Neogloboquadrina pacyderma* sin. (planktic foraminifera)	1860 ±55	1.34 – 1.22	1.41 – 1.13	1.27 ±0.66	0.58	1.10
AAR-12706	TTR13AT 455G, 67-68 cm	67-68	*Neogloboquadrina pacyderma* sin. (planktic foraminifera)	13510 ±90	15.7 – 15.3	15.8 – 15.2	15.5 ±0.2	0.33	1.82
AAR-9719	TTR13AT 455G, 73-74 cm	73-74	*Neogloboquadrina pacyderma* sin. (planktic foraminifera)	14700 ±90	17.4 – 17.1	17.5 – 17.0	17.2 ±0.2	0.33	1.82
AAR-10109	TTR13AT 455G, 100-103 cm	100-103	*Neogloboquadrina pacyderma* sin. (planktic foraminifera)	15630 ±75	18.4 – 18.1	18.5 – 18.0	18.3±0.2	0.01	-6.88
AAR-9720	TTR13AT 455G, 203-204 cm	203-204	*Neogloboquadrina pacyderma* sin. (planktic foraminifera)	17210 ±150	20.5 – 20.1	20.7 – 19.9	20.3 ±0.2	-0.18	2.58
AAR-9721	TTR13AT 455G, 304-305 cm	304-305	*Neogloboquadrina pacyderma* sin. (planktic foraminifera)	20050 ±150	23.4 – 23.0	23.7 – 22.9	23.3 ±0.2	0.05	2.73
AAR-9722	TTR13AT 455G, 399-400 cm	399-400	*Neogloboquadrina pacyderma* sin. (planktic foraminifera)	21250 ±160	25.4 – 25.0	25.5 – 24.7	25.1 ±0.2	-0.32	1.97
AAR-9723	TTR13AT 455G, 548-550 cm	548-550	*Neogloboquadrina pacyderma* sin. (planktic foraminifera)	25690 ±240	29.4 - 28.8	29.7 - 28.6	9.1 ±0.3	-0.50	1.19

Mössbauer spectroscopy and clay minerology data

Table S2. Clay mineralogy for core 455G. Mössbauer spectroscopy data for one Fe^{2+} and one Fe^{3+} quadrupoles reported as % Fe^{2+} in relation to the total Fe^{2+} and Fe^{3+} content. V = vermiculite, S = smectite.

Depth, m	% <2 µm	%Fe^{2+}	Clay mineral
0.40	33	35	V(+S)
0.50		37	
0.60		37	
0.70	25	27	S(+V)
0.80		33	
0.90		31	
1.00		32	
1.25	36	39	V+S
1.37		36	
1.62		37	
3.05	30	42	V+S
4.95	45	35	V+S
5.48	53	40	V(+S)

Foraminiferal assemblage data and ecological groups

A number of the species may be groups according to their main ecological preferences; groupings used for both cores 455G and 479G in this paper are, in order of appearance in Fig. 4:

- **Sea-ice species**: *Islandiella helenae, Stainforthia feylingi* (Seidenkrantz, 2013; Knudsen & Seidenkrantz, 1994).
- **Polar water species** (medium blue): *Cassidulina reniforme* (Polyak et al., 2002; Steinsund et al., 1994), may also tolerate chilled Atlantic water.
- **Chilled Atlantic water** (orange): *Islandiella norcrossi* (Rytter et al., 1994; Lloyd, 2006).
- **Atlantic water species** (red): *Cassidulina neoteretis* (Seidenkrantz, 1995), Miliolida.
- **Deep-water species** (NEADW, brown): *Astrononion echolsi, Epistominella exigua, Ioanella tumidula, Melonis pompilioides, Nuttallides umbonifera, Oridorsalis tenerus, Pullenia bulloides, Pullenia subcarinata, Pullenia simplex, Tosaia hanzawai* (e.g., Bilodeau et al., 1994).
- **High-energy species** (yellow green)**:** *Cibicides lobatulus, Discorbinella* spp. (mainly *Discorbinella araucana*), *Trifarina angulosa, Astrononion gallowayi, Astrononion stelligerum* (e.g. Murray, 1991). Note that *Cibicidoides wuellerstorfi* is not included in the high-energy group of Fig. 4 but is shown separately in Fig. 3.

- Tolerates **unstable conditions** (dark blue): *Elphidium clavatum* (Polyak et al., 2002; Steinsund et al., 1994).
- **High productivity** species (dark green): *Alabaminella weddellensis, Bolivina* spp., *Bulimina Nonionella turgida* group (includes *N. turgida digitata*), *Pullenia osloensis, Sagrina subspinescens, Stainforthia concava*, spp. (e.g. Murray, 1991).

The distribution of selected species from core 445G is shown in Fig. S3 while a list of the species mentioned in text and/or figures are listed with author names in Table S3.

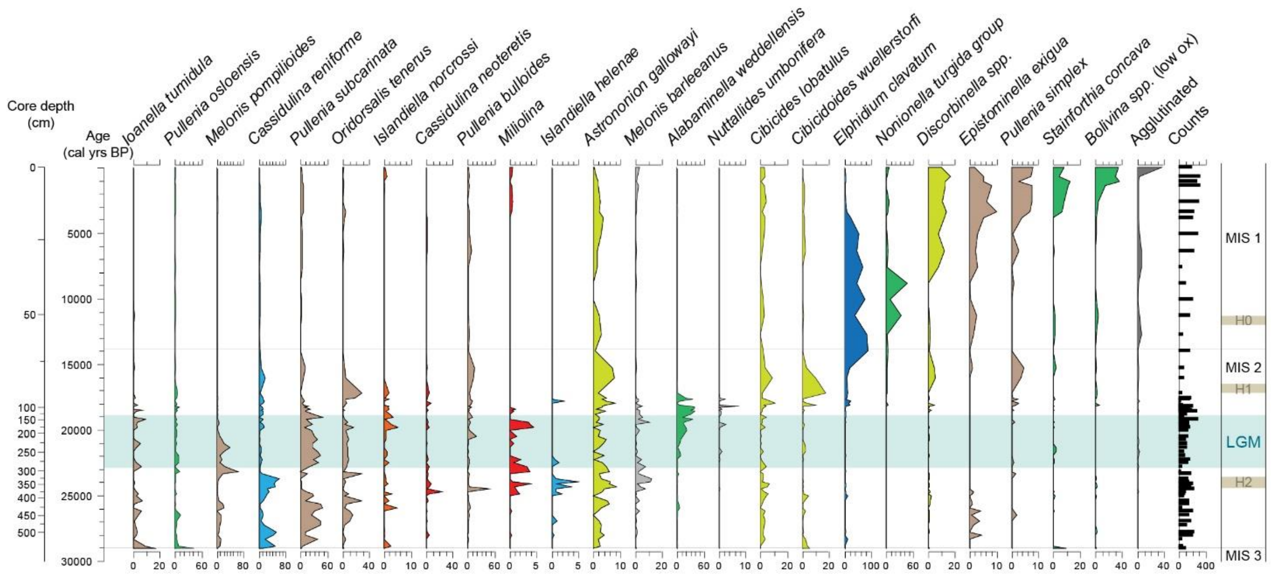

Figure S3. Selected benthic foraminifera through core TTR13-AT-455G versus age; data are shown as percentage distribution in relation to the total benthic foraminiferal assemblage. Colours according to the species' relation to ecological groups. Marine Isotope Stages (MIS) boundaries are based on Lisiecki & Raymo (2005), while the Last Glacial Maximum is defined as the period 23-19 kyr BP according to EPILOG (Mix et al., 2001) and MARGO (Kucera et al., 2005). The MIS 3/2 and MIS 2/1 boundaries are marked as gray horizontal lines, while the expected location of Heinrich events are marked in light brown.

Table S3. List of benthic foraminiferal species mentioned in text or figures.

**Benthic foraminiferal taxa**	*Melonis barleeanus* (Williamson, 1858)
Alabaminella weddellensis (Earland, 1936)	*Melonis pompilioides* (Fichtel & Moll, 1798)
Astrononion echolsi Kennett, 1967	Miliolida
Astrononion gallowayi Loeblich & Tappan, 1953	*Nonionella turgida* (Williamson, 1858)
Astrononion stelligerum (d'Orbigny, 1839)	*Nuttallides umbonifera* (Cushman, 1933)
Bolivina spp.	*Oridorsalis tenerus* (Brady, 1884)
Cassidulina neoteretis Seidenkrantz, 1995	*Pullenia bulloides* (d'Orbigny, 1846)
Cassidulina reniforme Nørvang, 1945	*Pullenia osloensis* Feyling-Hanssen, 1954
Cibicides lobatulus (Walker & Jacob, 1798)	*Pullenia simplex* Rhumbler, 1931
Cibicidoides wuellerstorfi (Schwager, 1866)	*Pullenia subcarinata* (d'Orbigny, 1839)
Discorbinella araucana (d'Orbigny, 1839)	*Stainforthia concava* (Höglund, 1947)
Discorbinella spp.	*Stainforthia feylingi* Knudsen & Seidenkrantz, 1994
Elphidium clavatum Cushman, 1930	*Tosaia hanzawai* Takayanagi, 1953
Epistominella exigua (Brady, 1884)	*Trifarina angulosa* (Williamson, 1858)
Ioanella tumidula (Brady, 1884)	**Planktic foraminiferal taxa**
Islandiella helenae Feyling-Hanssen & Buzas, 1976	*Neogloboquadrina pachyderma* (Ehrenberg, 1861)
Islandiella norcrossi (Cushman, 1933)	*Turborotalita quinqueloba* (Natland, 1938)

Extended discussion: Antarctic Bottom Water

Keigwin and Swift (2017) found evidence for previously unrecognized deep-water masses formed in the subpolar North Atlantic during the LGM. These authors conclude that these water masses probably originated from brine rejection at the Labrador Sea margin, where water masses of sufficient density were formed that could ventilate the deep western North Atlantic basin below a water depth of 4.2 km. In the deepest basins, these very deep waters of North Atlantic origin were overlain by slightly less dense Antarctic Bottom Water (AABW), which during parts of the glacial may have reached further north than observed today (Clauzet et al., 2007). However, during the LGM, these southern-sourced waters may not have been as spatially extensive as previously believed (Howe et al., 2016), and, in contrast to previous findings the northward spreading of AABW may have been very limited (Huang et al., 2020), coinciding with the sustained production of deep water NADW (Howe et al., 2016). None of our study sites reach water depth sufficient for tracking the present levels of the AABW, but if the AABW did indeed reach further north and to shallower depth as previously suggested, this could potentially be reflected in the data from core 455G. However, the benthic

foraminiferal assemblages here show a persistent, albeit varying, influx of NADW to the northernmost Labrador Sea. The near concurrent peaks in the two species *Alabaminella weddellensis* and *Nuttallides umbonifera* (Fig. S3) are notable, as these species have previously been considered to thrive in AABW (Schnitker, 1974; Corliss, 1979), but have later been shown to be cosmopolitan species (e.g., Carman & Keigwin, 2004; Sun et al., 2006), and cannot be used as indicators for AABW. Thus, although based on our data we cannot fully rule it out, we see no evidence of a relatively shallow AABW influx into the Labrador Sea.

References

Bilodeau, G., de Vernal, A. E. & Hillaire-Marcel, C. Benthic foraminiferal assemblages in Labrador Sea sediments: relations with deep-water mass changes since deglaciation. *Can. J. Earth Sci.* **31**, 128-138 (1994).

Bronk Ramsey, C. Bayesian analysis of radiocarbon dates. *Radiocarbon* **51**, 337–360 (2009).

Carman, M. R. & Keigwin, L. D., Preservation and color differences in Nuttallides umbonifera. *J. Foramin. Res.* **34** (2), p. 102–108 (2004).

Corliss, B. H. Quaternary Antarctic Bottom-Water history: Deep-sea benthonic foraminiferal evidence from the southeast Indian Ocean. *Quatern. Res.* **12** (2), 271-289, 10.1016/0033-5894(79)90062-0 (1979).

Clauzet, G., Wainer, I., Lazar, A., Brady, E. & Otto-Bliesner, B. A numerical study of the South Atlantic circulation at the Last Glacial Maximum. *Palaeogeogr., Palaeoclimatol., Palaeoecol.* **253**, 509–528, 10.1016/j.palaeo.2007.06.018 (2007).

Hemming, S. R. Heinrich events: Massive late Pleistocene detritus layers of the North Atlantic and their global climate imprint. *Rev. Geophysics* **42**, RG1005, 10.1029/2003RG000128 (2004).

Howe, J. N. W. *et al.* North Atlantic deep Water Production during the Last Glacial Maximum. *Nature Commun.* **7**, 11765, 10.1038/ncomms11765 (2016).

Huang, H., Gutjahr, M., Eisenhauer, A. & G. Kuhn, G. No detectable Weddell Sea Antarctic Bottom Water export during the Last and Penultimate Glacial Maximum. *Nature Commun.* **11**, 424, 10.1038/s41467-020-14302-3 (2020).

Jennings, A. E. *et al.* 2017. Ocean forcing of Ice Sheet retreat in central west Greenland from LGM to the early Holocene. *Earth Planet. Sci. Lett.* **472**, 1–13, 10.1016/j.epsl.2017.05.007.

Keigwin, L. D. & Swift, S. A. Carbon isotope evidence for a northern source of deep water in the glacial western North Atlantic. *PNAS* **114** (11), 2831-2835, 10.1073/pnas.1614693114 (2017).

Kenyon, N. H., Ivanov, M. K., Akhmetzhanov, A. M., Kozlova, E. V. & Mazzini, A. (Eds.), Interdisciplinary studies of North Atlantic and Labrador Sea Margin Architecture and Sedimentary Processes. *Intergovernm. Oceanograp. Comm. Technical Series* **68**, UNESCO, 92 pp (2004).

Knudsen, K. L. & Seidenkrantz, M.-S. *Stainforthia feylingi* new species from arctic to subarctic environments, previously recorded as *Stainforthia schreibersiana* (Czjzek). *Cushman Found. Foramin. Res. Spec. Publ.* **32**, 5–13 (1994).

Kucera, M., Rosell-Melé, A., Schneider, R., Waelbroeck, C. & Weinelt, M. Multiproxy Approach for the Reconstruction of the Glacial Ocean surface, *Quatern. Sci. Rev.* **24**, 7-9, 813-819 (2005).

Lisiecki, L. E. & Raymo, M. E. A Pliocene-Pleistocene stack of 57 globally distributed benthic δ18O records. *Paleoceanogr.* **20**, PA1003, https://doi.org/10.1029/2004PA001071 (2005).

Lloyd, J. M. Modern Distribution of Benthic Foraminifera From Disko Bugt, West Greenland. *J. Foramin. Res.* **36**, 315–331, 10.2113/gsjfr.36.4.315 (2006).

Lloyd, J. M. *et al.* A 100 yr record of ocean temperature control on the stability of Jakobshavn Isbrae, West Greenland. *Geology* **39** (9), 867-870, 10.1130/G32076.1 (2011).

Locarnini, R. A. *et al.* 2018. *World Ocean Atlas 2018, Volume 1: Temperature*. A. Mishonov Technical Ed.; NOAA Atlas NESDIS 81, 52 pp.

Mix, A. C., Bard, E. & Schneider, R. Environmental processes of the ice age: land, oceans, glaciers (EPILOG). *Quatern. Sci. Rev*. **20**, 627–658 (2001).

Murray J.W. 1991. Ecology and palaeoecology of benthic foraminifera. (Longman Scientific and Technical, Harlow, 1991).

Polyak, L. *et al*. Benthic foraminiferal assemblages from the southern Kara Sea, a river-influenced arctic marine environment. *J. Foramin. Res.* **32**, 252–73 (2002).

Reimer, P. J. *et al.* IntCal13 and Marine13 radiocarbon age calibration curves 0–50,000 years cal BP. *Radiocarbon* **55**, 1869–1887 (2013).

Rytter, F., Knudsen, K. L., Seidenkrantz, M.-S. & Eiríksson, J. Modern distribution of benthic foraminifera on the North Icelandic shelf and slope. J. *Foramin. Res*. **32**, 217–244 (2002).

Schnitker, D. West Atlantic abyssal circulation during the past 120,000 years: *Nature* **248**, 385–387 (1974).

Seidenkrantz, M.-S. *Cassidulina teretis* Tappan and *Cassidulina neoteretis* new species (Foraminifera): stratigraphic markers for deep sea and outer shelf areas. *J. Micropalaeontol.* **14**, 145–157 (1995).

Seidenkrantz, M.-S. Benthic foraminifera as palaeo sea-ice indicators in the subarctic realm – examples from the Labrador Sea–Baffin Bay region. *Quatern. Sci. Rev.* **79**, 135–144, 10.1016/j.quascirev.2013.03.014 (2013).

Sheldon, C. *et al.* Ice stream retreat following the LGM and onset of the west Greenland current in Uummannaq Trough, west Greenland. *Quatern. Sci. Rev.* **147**, 27–46, 10.1016/j.quascirev.2016.01.019 (2016).

Seidenkrantz, M.-S. *et al.* Southwest Greenland shelf glaciation during MIS 4 more extensive than during the Last Glacial Maximum. *Scient. Rep.* **9**, 15617, 10.1038/s41598-019-51983-3 (2019).

Steinsund, P.I., Polyak, L., Hald, M., Mikhailov, V. & Korsun, S. Distribution of calcareous benthic foraminifera in recent sediments of the Barents and Kara Sea. In: Steinsund, P. I. *Benthic Foraminifera in Surface Sediments of the Barents and Kara Seas: Modern and Late Quaternary Application*. Ph.D. thesis, Department of Geology, Institute of Biology and Geology, University of Tromsø, Norway (1994).

Zweng, M. M. *et al.*, 2018. *World Ocean Atlas 2018*, Volume 2: Salinity. A. Mishonov Technical Ed.; NOAA Atlas NESDIS 82, 50 pp.
